# Reference Values for Clinical Laboratory Parameters in Young Adults in Maputo, Mozambique

**DOI:** 10.1371/journal.pone.0097391

**Published:** 2014-05-14

**Authors:** Nelson Tembe, Orvalho Joaquim, Eunice Alfai, Nádia Sitoe, Edna Viegas, Eulalia Macovela, Emilia Gonçalves, Nafissa Osman, Sören Andersson, Ilesh Jani, Charlotta Nilsson

**Affiliations:** 1 Instituto Nacional de Saúde, Maputo, Mozambique; 2 Department of Laboratory Medicine, Karolinska Institutet, Huddinge, Sweden; 3 Faculty of Medicine, Eduardo Mondlane University, Maputo, Mozambique; 4 Hospital Central de Maputo, Maputo, Mozambique; 5 Örebro University Hospital, Örebro, Sweden; 6 Public Health Agency of Sweden, Solna, Sweden; 7 Department of Microbiology, Tumor and Cell Biology, Karolinska Institutet, Stockholm, Sweden; University of Utah School of Medicine, United States of America

## Abstract

**Background:**

Clinical laboratory reference values from North American and European populations are currently used in most Africans countries due to the absence of locally derived reference ranges, despite previous studies reporting significant differences between populations. Our aim was to define reference ranges for both genders in 18 to 24 year-old Mozambicans in preparation for clinical vaccine trials.

**Methods:**

A cross-sectional study including 257 volunteers (102 males and 155 females) between 18 and 24 years was performedat a youth clinic in Maputo, Mozambique. All volunteers were clinically healthy and human immunodeficiency virus, Hepatitis B virus and syphilis negative.Median and 95% reference ranges were calculated for immunological, hematological and chemistry parameters. Ranges were compared with those reported based on populations in other African countries and the US. The impact of applying US NIH Division of AIDS (DAIDS) toxicity tables was assessed.

**Results:**

The immunology ranges were comparable to those reported for the US and western Kenya.There were significant gender differences in CD4^+^ T cell values 713 cells/µL in males versus 824 cells/µL in females (p<0.0001). Hematologic values differed from the US values but were similar to reports of populations in western Kenya and Uganda. The lower and upper limits of the ranges for hemoglobin, hematocrit, red blood cells, white blood cells and lymphocytes were somewhat lower than those from these African countries. The chemistry values were comparable to US values, with few exceptions. The upper limits for ALT, AST, bilirubin, cholesterol and triglycerides were higher than those from the US. DAIDStables for adverse events predicted 297 adverse events and 159 (62%) of the volunteers would have been excluded.

**Conclusion:**

This study is the first to determine normal laboratory parameters in Mozambique. Our results underscore the necessity of establishing region-specific clinical reference ranges for proper patient management and safe conduct of clinical trials.

## Introduction

The number of clinical trials related to HIV/AIDS, tuberculosis and malaria conducted in Africa is increasing sharply and is expected to increase further in the coming years [1], [2]. Routine health assessment and management of clinical trials relies on accurate laboratory references. Of primary importance in vaccine clinical trials is the evaluation of safety and tolerability in a clinically “normal” population. Furthermore, volunteers may be assessed for disease progression and evaluation of possible clinical trial-associated toxicity and adverse events.

Laboratory reference intervals for healthy populations have not been established in most African countries. Common practice in these countries, including Mozambique, is to use reference ranges derived from populations living in Europe or the United States (US). Studies have shown differences between clinical reference ranges in African populations compared to those established in industrialized countries [3]–[6]. Several studies have also reported that laboratory parameters vary geographically by ethnic origin, genetics, gender, altitude and environmental factors [3], [4], [7]–[9].

The use of improper clinical reference ranges to assess participant eligibility and safety for clinical trials may lead to unnecessary exclusion of eligible participants, contribute to over-reporting of adverse events (AEs) [10] and increase the number of referrals for clinical investigations. Laboratory abnormalities based on non-indigenous laboratory parameters and medical abnormalities were reported to be the main reasons volunteers were excluded from two Kenyan HIV vaccine clinical trials [7]. Moreover, studies have suggested that use of the US NIH Division of AIDS (DAIDS) toxicity tables may not be appropriate for African populations [10], [11].

Prior to execution of a phase I/II HIV vaccine trial (TaMoVac 01) in Mozambique, we performed a study to define the prevalence and incidence of HIV and other sexually transmitted viruses in Maputo in a population of young adults. This was also an opportunity to establish clinical reference values in 18 to 24 year-old Mozambicans.

This study establishes reference ranges for immunological, hematological and chemistry parameters in healthy young adults in Mozambique. We determined gender differences and compared values established for Mozambican young adults with those previously reported for the same age group in other African countries and with established intervals from the US (Massachusetts General Hospital, MGH-USA) [12]. Additionally, we applied the division of AIDS (DAIDS) toxicity tables for grading of AEs [13] to evaluate their potential implications for vaccine trials.

## Materials and Methods

### Ethics Statement

This study was approved by the National Bioethics Committee for Health of Mozambique. Written informed consent was obtained from each participant prior to conducting any study procedures.

### Study site and subjects

The study took place at the SAAJ clinic (SAAJ: ‘Serviço Amigo de Adolescentes e Jovens’ or Adolescent and Youth Friendly Service) at the Department of Obstetrics and Gynecology, Maputo Central Hospital. The SAAJ clinic provides services (free of charge) for young people seeking counseling and treatment for any health problem, but with particular attention to reproductive health and control of sexually transmitted infections (STIs). A low prevalence of STIs and HIV and a high level of awareness has previously been reported in a study performed at the SAAJ clinic [14].

Maputo is the capital of Mozambique and is situated in a coastal area adjacent to the Indian Ocean, between the coordinates 25° 50′ and 26° 10′ S and 32° 30′ and 32° 40′ E [15]. The city is situated at an average altitude of 47 meters, has an area of 346.77 km^2^ and had a population of 1,094,315 in 2007 [16].

A total of 257 healthy individuals between 18 and 24 years old were recruited from a cohort of youths participating in a study of the prevalence and incidence of sexually transmitted viruses at the SAAJ clinic, Maputo Central Hospital. Medical staff performed physical examinations and collected clinical histories. Volunteers who were febrile, pregnant, or seropositive for HIV, syphilis or hepatitis B surface antigens were excluded from the study.

### HIV, syphilis and hepatitis B screening

The national algorithm for HIV testing was used to diagnose HIV. HIV testing was performed using two immunochromatographic assays, the Determine HIV-1/2 (Inverness Medical, Bedford, United Kingdom) followed by the UniGold HIV-1/2 (Trinity Biotech, Bray, Ireland). Syphilis testing was performed using SD Bioline Syphilis 3.0(Standard Diagnostics, Suwon City, South Korea). Serum samples were tested for Hepatitis B virus (HBV) using the Hepatitis B Surface Antigen (HBsAg) ELISA Kit (Human, Wiesbaden, Germany).

### Pregnancy testing

A urine pregnancy test was administered to all females prior to collection of blood samples, using the QuickVue One-Step HCG Urine Test (Quidel Corporation, San Diego, USA).

### Blood collection

Sample collection took place between August 2009 and September 2012. Most samples (70%) were collected between November 2009 and August 2010, but the inclusion period was extended to recruit additional males to the study. Blood was collected in 4 ml EDTA Vacutainer tubes (Becton-Dickinson, Franklin Lakes, New Jersey, USA)in preparation for lymphocyte and hematological testing. Whole blood was collected in 10 ml serum Vacutainer tubes (Becton-Dickinson, USA)in preparation for testing chemical parameters and HBV status. Samples were collected in the morning between 8.00 AM and 12.00 noon, kept at room temperature and transferred to the laboratory of the National Institute of Heath in Maputo for analysis.

### Flow cytometry analysis for Immunophenotyping

Immunophenotyping was performed using a FACS Calibur flow cytometer (Becton-Dickinson, Franklin Lakes, New Jersey, USA). Samples were analyzed within 24 h of specimen collection. In brief, 20 µl of CD3^FITC^/CD8^PE^/CD45^perCP^/CD4^APC^ or CD3^ FITC^/CD16+CD56^ PE^/CD45^PerCP^/CD19^ APC^ MultiTest reagents (Becton-Dickinson, USA) was mixed with 50 µl of whole blood and incubated in the dark at room temperature for 15 min. Red blood cells were then lysed by adding 450 µl of fluorescence-activated cell sorter lysing solution (Becton Dickinson, USA).The tubes were then incubated at room temperature for another 15 min. MultiSET software (Becton-Dickinson, USA) was used to perform the analysis.

### Hematological analysis

A complete blood count and differential was performed using the Sysmex KX-21N Hematology Analyzer (Sysmex Corporation, Kobe, Japan) as recommended by the manufacturer. The samples were analyzed within 6 h of specimen collection. The machine automatically dilutes a whole-blood sample, lyses and counts the cells, and then gives a printout result. Seventeen parameters were analyzed; leukocytes (WBC), erythrocytes (RBC), platelets (PLT), lymphocytes (LYM), neutrophils (NEUT), hemoglobin concentration (Hb), hematocrit (HCT), mean corpuscular volume (MCV), mean corpuscular hemoglobin (MCH), mean corpuscular hemoglobin concentration (MCHC), red blood cell distribution width measured by standard deviation (RDW-SD), red blood cell distribution width measured by coefficient of variation (RDW-CV), platelet distribution width (PDW), mean platelet volume (MPV), platelet larger cell ratio (P-LCR) and the percentages of lymphocytes (LYM), neutrophils (NEUT), and the mixed population of monocytes, basophiles and eosinophils (MXD). The absolute cell counts were expressed as number of cells × [10^6^] per liter.

### Biochemistry analysis

Serum chemistry was performed using a Vitalab Selectra Junior (Vital Scientific, Dieren, Netherlands) per the manufactureŕs instructions. Serum was separated within 4 h of collection and analyzed within 7 h of blood draw. Each sample was analyzed for creatinine, total bilirubin (T-Bil), albumin, aspartate aminotransferase (AST), alanine aminotransferase (ALT), glucose, urea, uric acid, amylase, HDL cholesterol, triglycerides and alkaline phosphatase (ALP).

### Quality control

In addition to commercial controls run daily with each instrument, the laboratory routinely participates (3 times per year) in external quality assurance testing programs distributed by Thistle (Thistle QA, Bryanston, South Africa) for hematology and clinical chemistry, and the International Quality Assurance Program-QASI (Public Health Agency of Canada, Ottawa, Canada) for lymphocyte typing. In the case of internal control failure, testing was suspended.

Clinical and laboratory personnel trained in GCP and GCLP, respectively, conducted the study according to ICH/GCP guidelines, employing standard operating procedures.

### Statistical analysis

Data was entered into Microsoft Office Excel 2007 and analyzed using R version 3.0.0 [17]. Prior to calculation of the reference ranges each parameter was check to be normally distributed using Shapiro-Wilks test; and if a deviation from normality was found the best Box-Cox power transformation was performed. Grubbs tests were performed to check for outliers and box plots and qqnorm plots were used to assess outliers visually. In general outliers were removed but their influence was checked by calculating the reference range with the outlier and comparing the reference range without the outlier.

Median and 95% reference ranges (2.5^th^- 97.5^th^ percentiles) were established for immunology, hematology and chemistry values. The non-parametric Wilcoxon rank-sum test (Mann-Whitney U test) was used to test for differences by gender. For all analysis the significance was set at 0.05.

For clinical laboratory parameters that did not meet the recommended minimum sample size of 120 individuals, which is recommended by the Clinical and Laboratory Standards Institute (CLSI) [18], percentiles were obtained from a boostrap procedure where 1000 resamples were performed per parameter and gender. On each resample, a percentile was calculated and the final percentile was the averages of the resampled percentiles [19], [20]. The p-values were obtained using a 10000 boostrap resamples per parameter to obtain the distribution of the Wilcoxon rank-sum test under the assumption of no difference between genders and then the original was compared to this distribution and area under curve on the right side of the histogram was considered as p-value.

Further outlier presence was checked using the plots of the resample of the mean minus the trimmed mean as explained by Chernick and Labudde [21] and performing the Silverman for multimodality on that plot. If multimodality was found the extreme value was removed from the analysis.

We then compared values established for Mozambican young adults with those previously reported for the same age group in other African countries [4], [11] and with established intervals from the US (the Massachusetts General Hospital, MGH-USA) [12]. Additionally, we applied the division of AIDS (DAIDS) toxicity tables for grading of adverse events [13] to evaluate their potential implication in vaccine trials.

## Results

A total of 257 healthy volunteers aged 18 to 24 years were enrolled in this study. Of these, 102 (40.3%) were males and 155 (59.7%) were females. The target of >120 males was not reached because of the comparatively low attendance of males at the Youth Clinic from which volunteers were recruited. The full range of lymphocyte subsets, hematology and chemistry analytes was not run in all subjects, primarily due to insufficient access to reagents required to test some parameters.

### Immunophenotyping

Table 1 shows the median values and reference ranges for lymphocyte subsets generated from participants in the study. Analyses of lymphocyte subsets revealed statistically significant differences between genders with respect to the absolute number of CD4^+^ cells, percentage of CD4^+^ cells, percentage of CD8^+^ cells, and CD4:CD8 ratios. Reference ranges for the absolute number of CD4^+^ cells (p<0.0001) and percentage of CD4^+^ cells (p<0.0001), CD4:CD8 ratios (p = 0.0001) as well asCD3+ cells (p = 0.0420) were significantly higher in females than males; however, the percentage of CD8^+^ cells was significantly higher in males (p = 0.0038). There were no significant gender differences in CD45^+^cell counts (total lymphocytes), percentage of CD3+ and absolute CD8^+^ cell count. Gender comparisons were also performed for CD16^+^56^+^ (natural killer cells) and CD19^+^ cells (B-cells) despite the comparatively low number of male samples analyzed (n = 29). A statistically significant gender difference was seen in percentage of CD16^+^56^+^ cells (p = 0.0390). No significant gender difference was found with respect to absolute number of CD16^+^56^+^ cells or absolute number and percentage of CD19^+^ cells.

**Table 1:** Lymphocytes subset reference ranges (median and 2.5^th^- 97.5^th^ percentiles) derived from young adults in Maputo city, Mozambique.

Parameter	Male		Female		Total		p
	N	Range	N	Range	N	Range	
CD45+ cells/µL	92	1958 (1185–3201)	135	2085 (1134–3678)	227	2046 (1151–3471)	0.0788
CD3+ cells/µL	91	1328 (716–1917)	129	1430 (756–2313)	220	1386 (729–2220)	0.0420
CD3+ T cells/µL (%)	92	66.8 (50.2–79.5)	133	67.8 (55.1–80.9)	225	67.5 (51.7–80.7)	0.2630
CD4+ T cells/µL	91	713 (357–1155)	135	824 (434–1479)	226	774 (381–1340)	<0.0001
CD4+ T cells/µL (%)	92	35.5 (23.2–49.1)	133	40.4 (29.9–53.7)	225	38.7 (25.8–52.2)	<0.0001
CD8+ T cells/µL	91	482 (214–902)	133	480 (234–965)	224	479 (218–952)	0.3954
CD8+ T cells/µL (%)	92	25.5 (14.9–41.5)	132	23.5 (14.7–34.5)	224	24.2 (14.7–37.6)	0.0038
CD4:CD8 ratio	91	1.5 (0.7–2.7)	133	1.7 (1.0–3.2)	224	1.6 (0.8–3.1)	0.0001
CD16+56+ cells/µL	29	351 (151–761)	101	333 (95–845)	130	338 (101–820)	0.3978
CD16+56+ cells/µL(%)	29	17.5 (10.3–36.0)	101	15.2 (4.1–31.1)	130	15.9 (4.9–34.5)	0.0390
CD19+ cells/µL	29	231 (97–475)	101	264 (85–548)	130	251 (86–545)	0.0751
CD19+ cells/µL (%)	29	12.3 (6.8–17.0)	100	12.3 (6.3–21.5)	129	12.3 (6.3–20.9)	0.2638

p-values indicate comparisons between males and females.

Our immune phenotype data were generally comparable to those derived from the North American populations provided by Becton-Dickinson or MGH-USA, although the lower and upper limits of the US reference ranges for all T cells were higher than those derived from the present study. The reference ranges for CD4^+^ and CD8^+^ T cells derived in the present study were more comparable to values reported from the population in western Kenya than in Uganda (Table 2).

**Table 2:** Lymphocyte reference ranges from youth in Maputo, Mozambique compared with sources from Africa and the United States of America.

Parameter	Maputo-Moz.	Western Kenya [11]	Uganda [4]	USA^a^
	(18–24 years old)	(18–34 years old)	(19–24 years old)	
**CD3 T-cells/µl**	729–2220	NA	NA	723–2737
**CD3 T-cells (%)**	51.7–80.7	NA	NA	56–86
**CD4 T-cells/µl**				
All	381–1340	444–1488	NA	404–1612
Male	357–1155	462–1306	504–1334	NA
Female	434–1479	440–1602	560–1961	NA
**CD4 T-cells (%)**				
All	25.8–52.2	NA	NA	33–58
Male	23.2–49.1	29–54	18.5–42.2	NA
Female	29.9–53.7	32–55	27.4–53.0	NA
**CD8 T-cells/µl**				
All	218–952	211–1078	NA	220–1129
Male	214–902	201–1104	286–1579	NA
Female	228–965	262–1167	151–1226	NA
**CD8 T-cells (%)**				
All	14.7–37.6	NA	NA	13–39
Male	14.9–41.5	14.9–44.0	12.7–41.7	
Female	14.7–34.5	17.5–35.0	9.2–29.5	
**CD4:CD8 ratio**	0.8–3.1	0.8–2.8	NA	NA
**B-cells**	86–545	NA	NA	80–616
**B-Cells (%)**	6.3–20.9	NA	NA	5–22
**Nk Cells**	101–820	NA	NA	84–724
**Nk Cells (%)**	4.9–34.5	NA	NA	5–26

a Reference ranges provided by Becton-Dickinson with the MultiTEST IMK Kit Reagent package.

NA – Not available.

### Hematology

Table 3 shows the medians and reference ranges for hematological parameters, grouped according to gender. We observed statistically significant differences between males and females in most hematological parameters, with the exception of PDW, MPV, absolute LYM, absolute MXD and percentage MXD. The males had higher values of RBC, Hb, HCT, MCV, MCH, MCHC and percentage LYM than females. The females had higher values of WBC, PLT, absolute NEUT and percentage NEUT, RDW-SD and RDW-CV than males.

**Table 3:** Hematology reference ranges (median and 2.5^th^- 97.5^th^percentiles) derived from young adults in Maputo, Mozambique.

Parameter	Male		Female		Total		P
	N	Range	N	Range	N	Range	
Hemoglobin (g/dL)	101	14.1 (12.3–16.4)	151	11.2 (7.0–13.1)	252	12.2 (7.5–15.8)	<0.0001
Hematocrit (%)	97	42.8 (25.2–50.4)	150	33.8 (19.5–40.3)	247	36.4 (20.2–47.2)	<0.0001
MCV (fL)	102	85.8 (72.4–92.9)	150	81.1 (59.1–94.3)	252	83.1 (63.0–94.1)	<0.0001
MCH (pg)	102	28.4 (22.2–47.7)	151	26.9 (16.4–48.0)	253	27.5 (18.0–48.7)	0.0063
MCHC (g/dL)	101	33.1 (30.4–54.2)	151	32.4 (25.8–56.1)	253	32.7 (28.1–55.9)	0.0143
RDW-SD (fL)	95	43.6 (37.0–50.0)	150	44.6 (36.3–52.6)	245	44.2 (36.4.–52.2)	0.0159
RDW-CV (%)	95	14.1 (11.6–18.6)	149	14.8 (12.2–23.5)	244	14.4 (11.9–23.4)	0.0084
PDW (fL)	97	13.5 (10.4–22.2)	147	13.5 (10.1–21.0)	244	13.5 (10.2–22.3)	0.8719
MPV (fL)	97	10.6 (8.7–13.1)	147	10.5 (8.5–12.7)	244	10.5 (8.5–13.0)	0.9564
Erythrocytes (10^6^/µL)	100	5.1 (2.7–6.1)	150	4.2 (2.3–5.0)	250	4.6 (2.4–5.9)	<0.0001
Platelets (10^3^/µL)	102	231.1 (116.2–392.1)	151	269.0 (128.8–503.0)	253	252.0 (125.2–488.0)	<0.0001
WBC (10^3^/µL)	101	4.6 (2.9–7.7)	151	5.6 (3.2–9.1)	252	5.1 (3.0–8.7)	<0.0001
Neutrophils (10^3^/µL)	101	2.4 (1.1–5.1)	149	3.2 (1.4–7.0)	250	2.7 (1.2–6.1)	<0.0001
Neutrophils (%)	95	52.5 (34.4–70.8)	146	57.1 (37.0–76.7)	241	54.7 (34.9–74.9)	0.0006
Lymphocytes (10^3^/µL)	97	1.8 (1.1–3.3)	150	1.9 (1.0–3.1)	247	1.9 (1.1–3.1)	0.0535
Lymphocytes (%)	97	39.1 (15.5–57.1)	150	35.1 (17.8–53.6)	247	37.0 (16.6–56.2)	0.0103
MXD (10^3^/µL)	91	0.3 (0.0–0.9)	132	0.3 (0.0–0.8)	223	0.3 (0.0–0.8)	0.3807
MXD (%)	85	6.8 (0.5–16.2)	131	6.3 (0.0–12.9)	216	6.3 (0.0–14.6)	0.1339

p-values indicate comparisons between males and females

Our reference ranges showed significant differences from the US population (Table 3). Close to 50% of our study participants had Hb values below the lower limits of the range determined in the US population; this was particularly true for females, 104 (69.3)% of whom had Hb values below the lower limit. We also found a considerable number of participants with platelet and WBC values outside the US reference ranges (47 (18%) and 64 (25%), respectively) (Table 4). Our reference ranges for young adults in Maputo were comparable to those derived from the western Kenyan and Ugandan populations. However, the lower and upper limits for some hematological parameters (Hb, HCT and lymphocytes, RBC and WBC)were lower than those derived from these African countries. The lower and upper limits of neutrophil and platelet reference ranges in our study were higher than those derived from populations in western Kenya and Uganda (Table 4).

**Table 4:** Comparison of hematology reference ranges derived from young adults in Maputo, Mozambique with those from other countries.

Parameter	Maputo-Moz	Western Kenya [11]	Uganda [4]	USA [12]
	(18–24 years old)	(18–34 years old)	(19–24 years old)	
**Hemoglobin (g/dL)**				
Male	12.3–16.0	11.4–16.9	11.5–17.1	13.5–17.5
Female	7.3–13.2	8.0–14.2	9.9–13.7	12.0–16.0
**Hematocrit (%)**				
Male	37.5–49.0	32.6–51.5	33.7–48.7	41.0–53.0
Female	20.9–40.2	23.2–44.3	28.9–40.0	36.0–46.0
**RBC's (10∧6 cells/µl)**				
Male	2.7–6.1	4.3–6.5	4.3–6.1	4.5–5.9
Female	2.3–5.0	3.4–5.7	3.6–5.4	4.0–5.2
**MCV (fl)**				
All	63.0–94.1	b 60–93	NA	80.0–100.0
Male	72.4–92.9	NA	67.2–91.8	NA
Female	59.1–94.3	NA	64.2–91.6	NA
**Platelets (10∧3 cells/µl)**				
All	125.2–488.0	b 103–390	NA	150–350
Male	116.2–392.1	102–307	98–306	NA
Female	128.8–503.0	88–439	95–368	NA
**WBC (10∧3 cells/µl)**				
All	3.0–8.7	b 3.3–9.3	3.7–9.7	4.5–11.0
Male	2.9–7.7	2.5–7.4	NA	NA
Female	3.2–9.1	3.3–9.7	NA	NA
**Neutrophils (10∧3 cells/µl)**				
All	1.2–6.1	b 0.9–5.2	1.0–3.5	1.8–7.7
Male	1.1–5.1	0.8–3.9	NA	NA
Female	1.4–7.0	1.3–5.4	NA	NA
**Lymphocytes (10∧3 cells/µl)**				
All	1.1–3.1	b 1.1–3.5	1.3–4.1	1.0–4.8
Male	1.1–3.3	1.0–3.5	NA	NA
Female	1.0–3.1	1.3–3.8	NA	NA

b values corresponding to individuals aged 13–34 years.

NA – Not available.

### Biochemistry

Table 5 shows the median values and reference ranges for clinical chemistry parameters. There was a statistically significant difference between genders in all clinical chemistry analytes, with the exception of triglycerides and amylase. Males had significantly higher levels of T-Bil, glucose, uric acid, AST, ALT, albumin, urea, creatinine, and ALP than females. Females had significantly higher levels of cholesterol (p = 0.0196) and HDL cholesterol (p = 0.0066) than males.

**Table 5:** Clinical chemistry reference values (median and 2.5^th^- 97.5^th^ percentiles) derived from young adults in Maputo, Mozambique.

Parameter	Male		Female		Total		P
	N	Range	N	Range	N	Range	
**Metabolism**							
Bilirubin, total (µmol/L)	98	12.1 (5.8–36.0)	154	7.5 (4.0–22.4)	252	9.0 (4.4–27.9)	<0.0001
Glucose (mmol/L)	96	4.4 (3.1–5.7)	155	4.1 (3.2–5.3)	251	4.2 (3.1–5.5)	0.0082
Triglycerides (mmol/L)	80	0.6 (0.3–1.5)	154	0.6 (0.3–1.4)	234	0.6 (0.3–1.5)	0.3386
Cholesterol (mmol/L)	84	3.6 (2.7–5.6)	152	3.9 (2.6–5.8)	236	3.8 (2.6–5.8)	0.0196
HDL Cholesterol (mmol/L)	63	1.3 (0.8–1.9)	152	1.4 (0.9–2.3)	215	1.4 (0.9–2.2)	0.0066
Uric Acid (mmol/L)	83	3.4 (1.7–5.0)	155	2.1 (1.0–3.3)	238	2.4 (1.1–4.4)	<0.0001
**Enzymes**							
ALT (U/L)	97	15.9 (6.5–53.2)	155	11.4 (4.8–38.5)	252	12.9 (5.0–48.2)	<0.0001
AST (U/L)	98	25.7 (16.8–45.5)	155	20.4 (13.5–37.0)	253	23.0 (13.7–42.8)	<0.0001
ALP (U/L)	83	157.4 (97.7–266.1)	148	135.3 (91.4–240.6)	231	142.6 (91.1–258.9)	<0.0001
Amylase (U/L)	79	91.9 (51.0–167.0)	154	89.2 (43.8–145.5)	233	89.8 (45.1–190.0)	0.3929
**Serum Proteins**							
Albumin (g/L)	84	49.7 (43.4–55.2)	151	47.7 (40.1–52.6)	235	48.4 (40.7–54.1)	<0.0001
**Kidney Function**							
Creatinine (µmol/L)	98	81.1 (58.2–109.0)	155	65.4 (45.0–86.6)	253	69.0 (47.1–103.2)	<0.0001
Urea (mmol/L)	84	3.8 (1.8–5.8)	153	2.8 (1.3–5.1)	237	3.1 (1.3–5.1)	<0.0001

p-values indicate comparisons between males and females.

The study reference ranges of clinical chemistry parameters were comparable to those derived from the North American population (MGH-USA), with a few exceptions. The upper limits of the reference ranges for ALT, AST, bilirubin and cholesterol derived in the present study were somewhat higher than those from the US. The Maputo values were lower compared to western Kenya in the same age group (Table 6). We also found a high proportion of study participants with glucose and T-Bil values outside of the US reference ranges, in 104 (41.1%) of 199 individuals and in 38 (15.0%) of 253 individuals, respectively (Table 7).

**Table 6:** Comparison of chemistry reference ranges derived from young adults in Maputo, Mozambique compared with those from western Kenya and the United States of America.

Analyte	Maputo-Moz	Western Kenya [11]	USA [12]
	(18–24 years old)	(18–34 years old)	
**METABOLISM**			
*Bilirubin, total (*µ*mol/L)*			
All	4.4–27.9	5.1–40.7	5.1–17.0
Male	5.8–36.0	5.3–50.7	NA
Female	4.0–22.5	5.8–36.1	NA
*Glucose (mmol/L)*	3.1–5.5	2.1–6.6	4.2–6.4
*Cholesterol (mmol/L)*	2.6–5.8	NA	<5.17
*Triglycerides (mmol/L)*	0.3–1.5	NA	<1.8
***ENZYMES***			
*ALT (U/L)*			
All	5.0–48.2	7.2–61.3	0–35
Male	6.5–53.2	12.0–80.6	NA
Female	4.8–38.5	10.7–61.3	NA
*AST (U/L)*			
All	13.7–42.8	13.8–50.4	0–35
Male	16.8–45.5	12.5–69.3	NA
Female	13.5–37.0	13.5–48.5	NA
*Amylase (U/L)*	43.5–160.4	NA	60–180
**SERUM PROTEIN**			
*Albumin (g/L)*	40.7–54.1	NA	35–55
**KIDNEY FUNCTION**			
*Creatinine (*µ*mol/L)*			
All	47.1–103.2	50–113	0–133
Male	58.2–109.0	54.2–137.8	NA
Female	45.0–86.6	52.4–96.8	NA

NA – Not available.

**Table 7:** Frequency of predicted adverse events in the Maputo youth cohort based on a comparison with values from DAIDS.

				Division of AIDS (DAIDS) toxicity grading							
		Number Ineligible									
		per US Comparison									
		Interval		Grade 1		Grade 2		Grade 3		Grade 4	
Parameter	N	n	%	n	%	n	%	n	%	n	%
**Hemoglobin (g/dL)**											
Male	100	14	14	1	0.9	0	0	0	0	0	0
Female	150	104	69.3	20	13.2	7	4.6	4	2.6	0	0
**Platelets (10∧6 cells/µl)**	253	47	18.6	3	1.2	4	1.6	0	0	0	0
**WBC (10∧6 cells/µl)**	252	64	25.4	1	0.4	0	0	0	0	0	0
**Neutrophils (10∧3 cells/µl)**	254	24	9.4	14	5.5	2	0.8	0	0	0	0
**Lymphocytes (10∧3 cells/µl)**	247	3	1.2	0	0	0	0	0	0	0	0
**T CD4 (Cells/µl)**	226	4	1.8	6	2.7	1	0.4	0	0	0	0
**ALT (U/L)**	253	15	5.9	22	8.7	5	1.9	0	0	0	0
**AST (U/L)**	253	14	5.5	145	57.3	52	20.6	0	0	0	0
**T-Bil (µmol/L)**	253	38	15.0	5	1.9	1	0.4	0	0	0	0
**Creatinine(µmol/L)**	253	0	0	0	0	0	0	0	0	0	0
**Glucose (mmol/L)**	251	104	41.1	0	0	0	0	0	0	0	0

### Implications for clinical trials

Table 7 shows the frequency of potential adverse events by applying the DAIDS toxicity grading, which is commonly used in clinical trials, to the values obtained from the cohort of young adults in Maputo. Among the hematologic parameters, Hb and neutrophil counts accounted for the majority of the abnormal classifications. The low Hb levels among our study participants would have resulted in 32 reported AEs; 21 (8.3%) as grade 1, 7 (4.6%) as grade 2 and 4 (2.6%) as grade 3. The low neutrophil counts would have resulted in 16 AEs: 14 (5.5%) as grade 1 and 2 (0.8%) as grade 2.With respect to the clinical chemistry parameters, ALT would have resulted in 27 AEs, 22 (8.7%) grade 1 and 5 (1.9%) grade 2 and AST would have resulted in 197 AEs, 145 (57.3%) grade 1 and 52 (20.6%) grade 2 (Table 7). Additionally, T-Bil values would have resulted in 6 reported AEs, 5 (1.9%) grade 1 and 1 (0.4%) grade 2. In total, based on all hematological and clinical chemistry parameters, 292 AEs are predicted to have been reported. Notably, 159 (62.8%) of the healthy volunteers included into the present study would have been excluded due to abnormal laboratory values should they have been evaluated for a clinical trial using US based criteria.

## Discussion

Clinical laboratory reference ranges have not previously been established in Mozambique. In this study we established reference ranges using samples derived from healthy young adults aged 18–24 years who attended a youth clinic at Maputo Central Hospital.

The number of males was disproportionate to the number of females (40.3% vs 59.7%), mainly because a lower proportion of males attended the SAAJ clinic. This affected the recruitment of males into the study, and the target of 120 subjects tested per analyte as recommended by CLSI [18] was not reached despite extending the enrollment period.However, a robust bootstrap analysis was used to eliminate bias due to the small sample size, as recommended by the Canadian laboratory initiative on pediatric reference intervals (CALIPER) [19].

Our immunophenotyping data showed significant gender differences between total lymphocyte counts, absolute T cell counts, absolute count of and percentage of CD4^+^ T cells and CD8^+^ T cell percentage, with females having higher values than males in each case. These findings are consistent with those reported for young adults in Kericho, Kenya [22]. Several other studies have also reported that females have higher CD4^+^ T cell counts than males [4], [23]–[26]. Overall, the reference ranges reported here for lymphocyte subsets were comparable to ranges reported for young adults in the US (Becton-Dickinson, USA) and western Kenya [11].

Due to insufficient access to reagents for natural killer cells and B cells determinations, only 34 males and 104 females were tested for these parameters. Although the number of participants was fewer than the 120 recommended by CLSI, a significant gender difference was seen for percentage of CD16^+^56^+^ natural killer cells and absolute count of CD19^+^ B cells. Lower percentages of CD16^+^56^+^ natural killer cells were seen in females compared to males, while absolute CD19^+^ B cells were lower in males than females. These findings were in general comparable to those reported in two studies performed in Tanzanian adults, which reported significant gender differences in percentage of natural killer cells and both the absolute count and percentage of CD19^+^ B cells [6], [25]. In both Tanzanian studies, females had a significantly lower percentage of natural killer cells than males, and males had a significantly lower absolute count and percentage of CD19^+^ B cells than females. These findings are consistent with a study of US adolescents (13–19 y) in which CD19^+^ B cells were reported to significantly decrease in males as age increased [24].

In the present study, we found significant gender differences in red blood parameters (RBC, HB, HCT and MCV), with males having higher values than females; these results are consistent with previous reports [3], [4], [5], [6], [11], [12], [27], [28], [29]. The reason for this gender difference may be due to the influence of androgen on erythropoiesis and menstrual blood loss in females [4], [11], [22]. Consistent with previous reports of African populations, a gender difference in platelet counts was seen, with females having higher values than males [11], [27], [30]. We also noted significant gender differences in total WBC count, again with females having higher values than males. This result is consistent with reports from Uganda [10], Ghana [24] and Kericho, Kenya [22] but differs from reports from Ethiopia [3], Central African Republic [5], and western Kenya [11], where total white blood cell counts did not vary between genders. In a UK study that included women of different ethnic origins (Caucasian, Afro-Caribbean and African) gender differences in total WBC and platelet counts were reported for all ethnic groups; women had higher values than men [31].

Most of the hematological values derived from this study were lower than those derived from the US population, which is consistent with studies of similar age groups conducted in western Kenya and Uganda [4], [11]. The lower limits for many parameters in the present study were lower than those derived from those two African countries. Notably, close to 50% of our study participants had Hb values that were outside the lower limits of the range derived from a US population, with 69.3% of females showing Hb values outside of the lower limit. Factors such as poor nutritional status, genetic red blood cell disorders or parasitic infections have been suggested to account for low Hb values. While a fairly low frequency of sickle cell trait (5.6%) was reported in an early study of pregnant women in Mozambique [32], a recent national survey of schistosomiasis and soil-transmitted helminthes showed that parasitic infections are common [33].

Clinical chemistry reference ranges derived in the present study were comparable to those derived from the North American population, with a few exceptions. The upper limits of the reference ranges for ALT, AST, Bilirubin and cholesterol derived in this study were slightly higher than those from the US, which is consistent with results from Uganda [10]. The Maputo reference values were lower than those reported in western Kenya for the same age group [11].

The clinical reference ranges derived from US MGH have been used in most clinical research studies due to the absence of local ranges. A comparison between values derived from the present study and US MGH values [17] reveals higher variations in most values, especially in hematology. If US MGH ranges were used as inclusion and/or exclusion criteria in potential clinical studies, 159 (62%) of the participants of the present study would be excluded due to abnormal laboratory parameters. However, if the local reference ranges were used, only 40 (16%) of the participants would be excluded. The use of improper reference ranges has been reported to affect the time period of trial enrollment due to the large number of participants that must be screened to reach the requested target sample size. The long enrollment period impacts both workload and study cost [10].

The Division of AIDS (DAIDS) toxicity table for grading adverse events is used in many clinical trials but may not be appropriate for the young adults included in the present study. For example, the lower limit ranges of neutrophil (1200 cells/µl), Hb (7.7 d/L) and AST (13.7 U/L) would qualify as grade 1, 3 and 1 AEs, respectively. The upper limits for ALT (48.2 U/L) and T-Bilirubin (27.9 µmol/L or 1.63 mg/dl) would be qualified as a grade 1 AEs. This is consistent with results found in Uganda [10] and western Kenya [11].

Several limitations are apparent in the design of the present study. Importantly, we did not screen for all medical conditions that might have influenced the laboratory findings. Participants with parasitic infections or with other subclinical conditions may have been included, which may have influenced the results. Secondly, the participants were recruited at a youth health center in Maputo and the findings may therefore not be generalizable to young adults in the rest of Mozambique or other African countries. Despite these limitations, the reference values established here are being used in ongoing HIV-vaccine trials in Maputo that recruit healthy volunteers from the cohort of young adults attending the Maputo Central Hospital Youth Clinic. Additionally, given that indigenous reference values have not been previously defined for any Mozambican population, the ranges defined in this study are suitable for use in assessment of young adults in a more generalized setting.

In conclusion, this study is the first to assess clinical laboratory reference ranges in Mozambique. The hematological and biochemistry reference values from this African population differ from those derived from a North American population. This study also highlights the need for region-specific clinical reference ranges for patient management and clinical research.
